# Dietary intake effects on severity of cancer treatment‐induced mucositis: A cross‐sectional study

**DOI:** 10.1002/hsr2.1706

**Published:** 2023-11-15

**Authors:** Soheila Manifar, Arghavan Tonkaboni, Aysan Sobhanifar, Kimia HafeziMotlagh, Sama Bitarafan, Mina Mazani, Paolo Bossi

**Affiliations:** ^1^ Department of Oral Medicine, School of Dentistry Tehran University of Medical Sciences Tehran Iran; ^2^ Cancer Research Center Tehran Iran; ^3^ Medical‐Surgical Oral Pathology Research Group University of Santiago de Compostela Santiago de Compostela Spain; ^4^ School of Dentistry, International Campus Tehran University of Medical Sciences Tehran Iran; ^5^ Iranian Center of Neurological Research, Neuroscience Institute Tehran University of Medical Sciences Tehran Iran; ^6^ FHMS Clinic, Neurology Department, Burnaby Hospital University of British Columbia Vancouver British Columbia Canada; ^7^ Medical Oncology Unit University of Brescia Brescia Italy

**Keywords:** cancer, chemotherapy, diet, mucositis

## Abstract

**Background and Aims:**

Oral mucositis is one of the most serious complications due to chemotherapy and radiotherapy in head and neck cancer treatment. Oral mucositis causes a wide range of clinical signs and symptoms, such as ulcers, pain, and dysphagia. Additionally, because of speech limitations, patients' self‐esteem will decrease, ultimately causing reduced quality of life. The primary objective of this study was to investigate the role of diet in the onset and progress of mucositis induced by chemotherapy and radiation therapy in patients with cancers.

**Methods:**

In this study, 121 patients with a mean age of 51.43 ± 13.08 years were selected randomly and referred to the cancer institute, where they underwent their first phase of chemotherapy. In this step, patients were examined and their severity of oral mucositis was graded according to the World Health Organization criteria. They completed a 3‐day allergen food recall and dietary recommendations were met. After completing the forms, four questionnaires were filled out for each patient, the patient's nutrition was analyzed using the N4 software, and the amount of macro‐ and micronutrients was measured.

**Results:**

Micronutrients such as aspartic acid, glycine, serine, proline, alanine, arginine, glutamic acid, and vitamin B12 and macronutrients such as rose water, sausage, beverages, coffee, and lamb meat were examined, and a significant difference was observed between groups (grade 1 and 2 mucositis) (*p* < 0.005). In patients with grade 2 mucositis, a lower level of vitamin B12 was reported (*p* < 0.005). There is a negative correlation between amounts of macro‐ and micronutrients and grades of oral mucositis.

**Conclusion:**

It can be concluded that diet plays a considerable role in the severity of oral mucositis caused by cancer treatment.

## 1 INTRODUCTION

Head and neck cancer is the sixth most common cancer globally and has become a global health concern.^1^ Various treatment modalities are recommended for cancer patients, such as surgery, radiation therapy, chemotherapy, or a combination thereof, and long‐term follow‐up.^2^ Chemotherapy and radiation therapy are nonsurgical methods used today to treat head and neck cancers, prolong survival rates, or completely cure the mass. These treatments have side effects that can interfere with the patient's quality of life. The most common complication observed is oral mucositis, which affects 75% of high‐risk patients receiving radiation or high‐dose chemotherapy.^3^

Clinically, oral mucositis includes various manifestations, from mucosal erythema to oral ulcers, which is the most common classification of oral mucosa by the World Health Organization (WHO) (Table 1).^4^ Mucositis complications include pain, dysphagia, dysgeusia, opportunistic infections, inability to eat, and difficulty breathing, ultimately decreasing the patient's quality of life. Chemotherapy‐induced mucositis initially occurs on keratinized mucosal surfaces, including the soft palate, tongue's ventral surface, mouth floor, and buccal and labial mucosa.^5^

**Table 1:** World Health Organization oral toxicity scale.

Grade	Anatomical, symptomatic, and functional components of oral mucositis
0	None
1	Soreness ± erythema
2	Erythema, ulcers, and patients cannot swallow solid food
3	Ulcers with extensive erythema and patients cannot swallow food
4	Mucositis to the extent that alimentation is not possible (TPN)

Abbreviation: TPN, total parenteral nutrition.

Oral mucositis caused by chemotherapy has significant differences from radiotherapy‐induced mucositis. Symptoms in chemotherapy patients begin 1 week after treatment and usually improve within 2 weeks. Meanwhile, mucositis caused by radiotherapy needs more time to heal and the wounds appear 2 weeks after the start of radiotherapy. They will heal 3–4 weeks after the end of the radiotherapy period.^6^ Oral mucositis risk factors can generally be classified into treatment and patient‐related factors. The dose and regimen of chemotherapy and radiation, the type of selected drugs, advanced age, patient's body mass index, and genetic predisposition are all risk factors for mucositis.^7^

The underlying mechanism of mucositis follows a specific step‐by‐step pattern. First, chemotherapy directly destroys the DNA of basal epithelial cells and produces reactive oxygen species that lead to the destruction of connective tissue. This process activates several factors in transcription, including kappa B factors, and p53 and their molecular pathway. This pathway of activation creates positive feedback and this cycle continues. Eventually, cell death or apoptosis occurs, which causes the clinical manifestations of mucositis with the atrophy of the mucous membrane.^4^

Currently, the care of patients with mucositis mainly includes palliative and supportive treatments, proper oral hygiene, a diet that does not stimulate mucus, avoiding acidic substances, and oral care products such as oral rinses and anesthetics for their analgesic effects. Research has shown that benzydamine can help prevent mucositis caused by chemotherapy.^8^ Some studies also support the potential role of vitamin A and E supplements in improving severe oral mucositis in patients undergoing chemotherapy. Advances in mucositis prevention and treatment improve quality of life while lowering health‐care expenses. Furthermore, better mucositis management can lead to better implementation of cancer therapy procedures, which are now more mucotoxic but may improve overall patient recovery.

There are limited studies on the effectiveness of food diaries in reducing the severity of oral mucositis in patients with head and neck cancer. In addition, the results have shown that there are still many controversies about the relationship between different types of food and the severity of oral mucositis, and more research is needed to generalize the results. This study aimed to investigate the relationship between the diet of patients undergoing chemotherapy and the severity of mucositis in patients referred to Imam Khomeini Hospital.

## 2 MATERIALS AND METHODS

One hundred and twenty‐one patients with breast, ovary, colon, liver, lung, stomach, pancreas, lymphoma, melanoma, osteosarcoma, prostate, squamous cell carcinoma (SCC), and leiomyosarcoma cancers who visited the cancer department of the hospital daily and were at the start of the cancer treatment phase were selected randomly.

According to the nutritionist, two questionnaires were prepared for each patient, consisting of a table of allergens for 1 week and a 24‐h food reminder for 3 days. Each food recall questionnaire included six nutritional meals: breakfast, snack, lunch, snack, dinner, and after dinner. The patients underwent a complete oral and dental examination by an oral medicine specialist. The degree of mucositis was determined according to the WHO standards. They were asked to complete a 3‐day questionnaire and a questionnaire on allergen nutrients and recommendations. After completing the forms, four completed questionnaires were obtained from each patient.

*Inclusion criteria*: Age over 12 years, no history of diseases that prevent the correct recall of diet, suffering from oral mucositis, and suffering from any type of cancer due to which the patient has been treated with chemotherapy or radiotherapy.

*Exclusion criteria*: Age less than 12 years old, the presence of psychotic diseases and diseases leading to lack of memory, such as Alzheimer's, and patients who have already been treated with radiotherapy.

The sample size for this study was determined to be 121 patients after considering that the food reminder questionnaire was completed during six different meals, the presence of 12 main independent variables in the regression model, and the main contextual, independent, and nonnutritional variables.

This study was approved by the Ethics Committee of Tehran University of Medical Sciences under the identification code of IR.TUMS.DENTISTRY.REC.1396.4161.

### 2.1 Statistical analysis

The diet of patients was analyzed using the N4 software and the intake of macro‐ and micronutrients was obtained. Afterwards, the relationship between nutritional intake and the severity of oral mucositis (grades 1–4) was investigated. We used SPSS software v.22.0 (IBM Corp) to analyze the regression model.

## 3 RESULTS

The patients in this study included 70 women (57.85%) and 51 men (42.15%) with cancer. The mean age of the patients was (51.43 ± 13.08) years. After the examination by an oral medicine specialist, participants were placed into two groups. Thirty‐two patients (24.44%) were in group 1 (grade 1 mucositis) and 87 patients (71.90%) were in group 2 (grade 2 mucositis).

Among the 16 types of cancer, the greatest number of patients with grade 1 mucositis belonged to the breast cancer group, with 13 (10.74%) people, and the most people with grade 2 mucositis belonged to the colon cancer group, with 23 (23%) people. The largest population with grade 1 and 2 mucositis is in the breast cancer group with 30 people (24.79), and among those with ovarian, melanoma, pancreatic, Kaposi sarcoma, esophagus cancer, SCC, and osteosarcoma, grade 1 mucositis was not reported.

According to the analyses conducted by the nutritionist and statistical consultant, it was found that a significant difference was observed between group 1 (grade 1 mucositis) and group 2 (grade 2 mucositis) (*p* < 0.005) (Table 2) considering the micronutrients such as amino acids such as aspartic acid, glycine, proline, alanine, arginine, glutamic acid, and vitamin b12 and macronutrients such as rose water, sausage, soft drinks, coffee, and beef.

**Table 2:** Patient characteristics.

Gender	Number	Percentage	Group 1	Group 2
Female	71	58.7	25 Patients	44 Patients
Male	50	41.3	5 Patients	45 Patients

The results of this study showed that the amount of consumption of all amino acids such as aspartic acid, glycine, proline, alanine, arginine, and glutamic acid was higher in group 2 (grade 2 mucositis) than in group 1 (grade 1 mucositis), but in the case of vitamin B12, this relationship was reversed in such a way that the amount of this vitamin in patients with mucositis grade 2 was lower than in group 1 (*p* < 0.005).

## 4 DISCUSSION

This study presents the effect of different dietary intakes of patients with severe chemotherapy‐induced oral mucositis in patients with various cancers. According to previous studies, several side effects are associated with chemotherapy and radiotherapy, such as oral mucositis, dysphagia, xerostomia, and weight loss. Consequently, these complications can lead to the cessation of radiotherapy and chemotherapy, which worsens the patient's overall status.^9^ However, the debate continues about the best strategies for effectively managing and treating mucositis induced by chemotherapy and radiotherapy.^10^

In a study by Salarvand et al., among 75 patients who received chemotherapy for breast cancer with a mean age of 47.2 ± 8, 81.3% of patients suffered from oral mucositis, and 52.4% of them showed grade 2 of WHO classification. They reported oral mucositis as the most common side effect of chemotherapy, negatively affecting the quality of life.^11^ Additionally, Nishimura et al. demonstrated that among 227 patients who received chemotherapy for various cancers, oral mucositis was highest in patients with breast cancer, followed by head and neck cancer. Furthermore, they concluded that mucositis is associated with gastrointestinal disturbances, anorexia, diarrhea, and dysphagia, which impair the patient's quality of life.^12^

The present study is the first study that specifically investigated the intake of amino acids and its comparison in patients with oral mucositis. One of the critical amino acids in mucositis is glutamine, the most abundant free amino acid in blood circulation. The importance of glutamine is in intracellular metabolism as it acts as a rich source of nitrogen and plays a crucial role in maintaining the structural integrity of the mucous membranes of the oral cavity and gastrointestinal tract.^13^ Among all the amino acids that have been considered to prevent or improve oral mucositis, most research has been done regarding glutamine.

The effect of glutamine on chemotherapy‐induced oral mucositis in esophageal cancer patients was investigated in a study by Tanaka and colleagues.^14^ Patients were divided into two groups: those receiving glutamine and those receiving glutamine plus elemental diet, and oral administration of glutamine and elemental diet started 1 week before chemotherapy and continued during treatment. This study showed that the incidence of grade ≥2 oral mucositis was highest in the control group (60%), followed by the glutamine group, and 10% in the glutamine plus elemental diet group. The results of our study indicate that the intake of this amino acid is higher in patients with grade 2 mucositis, and there is a significant difference between the two groups (*p* < 0.005) (Table 3).

**Table 3:** Micronutrients and macronutrients, which has a significant difference between group 1 and group 2 (*p* < 0.05).

Nutrient	Groups	Mean intake amount	*p* Value
Aspartic acid	1	107.77 ± 54.34	0.010
	2	337.59 ± 135.02	
Glycine	1	35.21 ± 17.79	0.006
	2	171.478 ± 71.11	
Serine	1	51.979 ± 23.97	0.005
	2	179.234 ± 84.59	
Vitamin B12	1	0.734 ± 0.070	0.011
	2	0.132 ± 1.261	
Glutamine	1	257.365 ± 122.27	0.008
	2	946.224 ± 425.16	
Proline	1	90.84 ± 45.93	0.017
	2	311.917 ± 136.63	
Alanine	1	40.359 ± 20.87	0.007
	2	208.990 ± 85.57	
Arginine	1	43.897 ± 20.87	0.007
	2	208.990 ± 85.57	
Rose water	1	0.0254 ± 0.07	0.013
	2	0.704 ± 0.29	
Sausage	1	00.0	0.045
	2	0.206 ± 0.04	
Beverage	1	0.305 ± 0.10	0.004
	2	2.259 ± 0.82	
Coffee	1	0.00	0.043
	2	0.868 ± 0.19	
Lamb meat	1	1.333 ± 0.43	0.008
	2	3.347 ± 1.59	

In a similar study, Tsujimoto and colleagues investigated whether l‐glutamine (glutamine) declines the severity of mucositis induced by chemoradiotherapy in head and neck cancer. This study was a double‐blind randomized controlled trial that included 40 patients with SCC. Results showed that glutamine could significantly eliminate the maximal mucositis grade and pain score at Weeks 4, 5, and 6.^15^ However, these results are somewhat in conflict with the results of the study done by Gliwska et al.,^16^ which determine whether glutamine supplementation is effective in reducing the incidence and severity of radiation‐ or chemoradiation‐induced mucositis and dermatitis in head and neck cancer patients. The incidence and severity of oral mucositis in the sixth week did not exhibit statistically significant differences between the two groups. However, due to the short follow‐up time and an insufficient number of samples, the results of this study should be interpreted with caution.

Arginine, alanine, and proline are other critical amino acids investigated in this study. The values of these three amino acids showed a significant difference between the two studied groups (*p* < 0.005). Arginine is one of the essential amino acids in the human body, made by citrulline. It plays a role in the secretion of hormones, regulation of immune activities, and healing of wounds. Arginine is also a precursor of proline, which is essential in the production of collagen and the recovery of mucosal wounds.^17^

Recent studies support the potential effects of the topical application of collagen precursor amino acids in mucosal wound healing. Researchers hypothesize that local administration of amino acids to treat mucosal wounds can benefit stromal vascularization and collagen deposition. Stimulating type II collagen synthesis occurs when different glycine, proline, and lysine amino acid concentrations are applied topically.^18^

Another critical micronutrient in cell regeneration and tissue repair is vitamin B12, which plays a crucial role in DNA synthesis. One of the primary studies to investigate the comparative effects of vitamin B12 and folate was done by Branda et al., which describes the effects of vitamin B12, folate, and dietary supplements on chemotherapy‐induced mucositis in breast cancer. Patients were asked to complete a questionnaire documenting their use of nutritional supplements. Before and after the first cycle of chemotherapy, blood samples were analyzed to assess serum vitamin B12 and folate levels, and complete blood counts were performed weekly. No significant association was found between oral mucositis and age, vitamin B12 levels, and folic acid levels.^13^ This study's results conflict with a study conducted by Chen et al.,^14^ which concluded that lower serum vitamin status could predict the severity of oral mucositis in patients with nasopharyngeal carcinoma. Their results are consistent with the results of our study that the amount of vitamin B12 consumption was reported to be lower in the grade 2 mucositis group (*p* < 0.005).

With this in mind, the European Society for Nutrition and Metabolism guideline states that early screening and assurance about patients' nutritional interventions is crucial in overcoming cancer‐related malnutrition.^15^ Prevention and treatment of oral mucositis are essential as malnutrition affects the efficacy of cancer treatment, prognosis, hospitalization period, and the patient's quality of life.^16^, ^19^ Early detection and treatment of oral SCC will contribute to a complete cure and 5‐year survival in 85%.^20^

The teamwork and support of the various specialists responsible for the care of cancer patients enable the tailoring of nutritional intervention to the clinical situation and the treatment of early toxicity and disease development.

## 5 CONCLUSION

Patients with all types of cancers should be consulted with a nutrition counselor before and during treatment. The patient also needs to be aware of the importance of nutrition and its role in developing mucositis during their treatment. It seems a great benefit to make a list of valuable and nutritious foods provided for them with an alert on allergens that have a potential role in mucositis.

## AUTHOR CONTRIBUTIONS

**Soheila Manifar**: Conceptualization; data curation; project administration; supervision. **Arghavan Tonkaboni**: Conceptualization; methodology; project administration; writing—original draft. **Aysan Sobhanifar**: Data curation; investigation; methodology. **Kimia HafeziMotlagh**: Data curation; writing—original draft; writing—review and editing. **Sama Bitarafan**: Data curation; visualization. **Mina Mazani**: Data curation; methodology; visualization. **Paolo Bossi**: Project administration; supervision; visualization.

## CONFLICT OF INTEREST STATEMENT

The authors declare no conflict of interest.

## TRANSPARENCY STATEMENT

The lead author Arghavan Tonkaboni affirms that this manuscript is an honest, accurate, and transparent account of the study being reported; that no important aspects of the study have been omitted; and that any discrepancies from the study as planned (and, if relevant, registered) have been explained.

## Data Availability

The data supporting this study's findings are available on request from the corresponding author. All authors have read and approved the final version of the manuscript and the corresponding author had full access to all of the data in this study and takes complete responsibility for the integrity of the data and the accuracy of the data analysis.
